# Seasonal dynamics of ammonia-oxidizing bacteria but not archaea influence soil nitrogen cycling in a semi-arid agricultural soil

**DOI:** 10.1038/s41598-022-10711-0

**Published:** 2022-05-04

**Authors:** L. M. Fisk, L. Barton, L. D. Maccarone, S. N. Jenkins, D. V. Murphy

**Affiliations:** SoilsWest, UWA School of Agriculture and Environment, The University of Western Australia, 35 Stirling Highway, Crawley, WA 6009 Australia

**Keywords:** Agroecology, Climate-change ecology, Biogeochemistry

## Abstract

Nitrification, a key pathway of nitrogen (N) loss from agricultural soils, is performed by ammonia-oxidizing bacteria (AOB) and archaea (AOA). We examined the seasonal dynamics (2 years) of ammonia oxidizer gene abundances across a gradient of soil carbon (C) and N in a semi-arid soil after 8 years of tillage and crop residue treatments. AOB was more dominant than AOA in the surface soil, as AOA were undetected in 96% of samples. Seasonal variation in AOB abundance was related to substrate availability; AOB gene copy numbers increased at the end of the growing season (during summer fallow) following higher concentrations in dissolved organic matter soil water. This suggests increased co-location between AOB and substrate resources in pores still filled with water as the soils dried. AOB was however not statistically related to soil ammonium concentrations, soil water content, rainfall or temperature. Organic matter inputs enhanced AOB abundance independent of seasonal variation. AOB abundance was greatest in autumn and immediately preceding the start of the growing season, and coincided with elevated soil nitrate concentrations. The growth of the AOB population is likely to contribute to increased risk of N loss through leaching and/or denitrification at the start of the crop growing season following summer fallow.

## Introduction

Nitrification plays a key role in nitrogen (N) loss processes, as the gatekeeper between internal soil N cycling and external loss by nitrate (NO_3_^−^) leaching to groundwater and nitrous oxide (N_2_O) emissions via nitrification and denitrification^1^. Nitrification has traditionally been considered a two-step process consisting of ammonia oxidation, followed by nitrite oxidation to form NO_3_^−^. Ammonia oxidation, the limiting step of nitrification, is performed by bacterial and archaeal ammonia oxidizers, which convert ammonium (NH_4_^+^) to nitrite^2,3^. Nitrifying bacteria from the genus *Nitrospira* species that are able to oxidise ammonia to NO_3_^−^ in a single step (‘comammox’) have also been recently identified in engineered and natural systems^4–6^, however their abundance and activity in agricultural soils currently appears limited^7,8^. Ammonia-oxidizing bacteria (AOB) belong to the beta- and gamma-subclasses of Proteobacteria, while ammonia-oxidizing archaea (AOA) belong to the phylum Thaumarchaeota^9,10^. Both AOA and AOB express functionally similar genes for the primary enzyme catalysing ammonia oxidation, ammonia monooxygenase (*amoA*)^9,11^. However, bacterial and archaeal ammonia oxidizers differ in their genetics, physiologies and metabolic processes, so are likely to differ in their adaptations to biotic and abiotic soil conditions (i.e. niche specialization) and utilization of resources (i.e. niche differentiation)^2,12^.

Niche specialization and differentiation between AOA and AOB are not yet clearly defined, but may be regulated by factors including soil pH, NH_4_^+^ supply, differences in requirements for copper or iron as enzyme constituents, or relative ability to carry out mixotrophic or heterotrophic growth^12–14^. Low soil pH is often reported to differentiate between AOA and AOB: at pH < 5.5 acidophilic AOA appear to be the dominant ammonia oxidizers, particularly of the phylogenetic cluster associated with *Nitrosotalea devanaterra*^15,16^. Although AOB abundance has been similar to AOA at pH as low as 4.5 in calcium chloride^17^, in that case it was unclear if AOB or AOA were responsible for ammonia oxidation. Low NH_4_^+^ substrate conditions may also allow AOA to dominate over AOB, as AOA have greater substrate affinity for NH_4_^+^ than AOB, and are more sensitive than AOB to inhibition by high concentrations of ammonia^18–20^. However, some AOA seem to be tolerant to high NH_4_^+^ concentrations^21^, with a meta-analysis of agricultural soils demonstrating N fertilizer form and soil pH affecting the response of AOA to N fertilization^20^.

Factors that regulate temporal variation in populations are often different from factors that regulate spatial fluctuations of populations^22^. Temporal variation in ammonia oxidizer gene abundance may be regulated by season (e.g. temperature and rainfall), soil organic matter (OM), and changes in NH_4_^+^ availability due to N fertilizer additions and periods of soil OM mineralization^8,23–25^. Taylor et al.^24^ speculated that increases of AOA *amoA* abundance (but not AOB) in spring of a fallow Oregon agricultural soil (with a dry-summer subtropical climate) was due to their utilization of another energy-generating metabolism besides ammonia oxidation (i.e. heterotrophy or mixotrophy). These authors also suggested that increased AOB *amoA* gene abundance in cropped soil at the same site was in response to increased NH_4_^+^ availability from N fertilizer additions^24^. In a semi-arid soil in Israel, greater AOA abundance than AOB in summer was hypothesized to be due to low NH_4_^+^ availability, as a consequence of low soil water content causing low mineralization rates^26^. Ammonia-oxidizing bacteria in the same soil may have also been limited by elevated temperature to a greater degree than AOA: in winter AOB shifted to greater dominance than AOA in the same soil^26^. However in agricultural soils, seasonal changes in the relative abundance of AOA to AOB may also be attributed to changes in nutrient availability in response to crop growth^8^.

The effect of soil OM content on *amoA* gene abundance of ammonia oxidizing bacteria and archaea varies. Many archaeal ammonia oxidizers appear to be able to take up organic C as an energy source (show heterotrophic and mixotrophic growth), so would be expected to be more abundant in soils with greater OM contents^27^. Bacterial ammonia oxidizers are limited to autotrophic growth, but increased supply of ammonia substrate from enhanced mineralization in higher OM soil might also increase AOB population size. However, studies have observed that increased OM or increased soil C and N pools have, for example, a greater AOA:AOB ratio^28^; no effect on AOB gene abundance^29,30^; or are negatively correlated to AOA gene abundance^30^. Temporal effects can further interact with spatial effects, such as an observed doubling of AOB gene abundance at the start of the growing season due to organic inputs that increased soil C and N pools, but not at other times of the year^31^; and a negative correlation between AOB but not AOA abundance and soil OM content changes with season^26^. These temporal changes may be due to the changes in soil water content and subsequent OM mineralization and NH_4_^+^ availability^26^.

**Figure 1:**
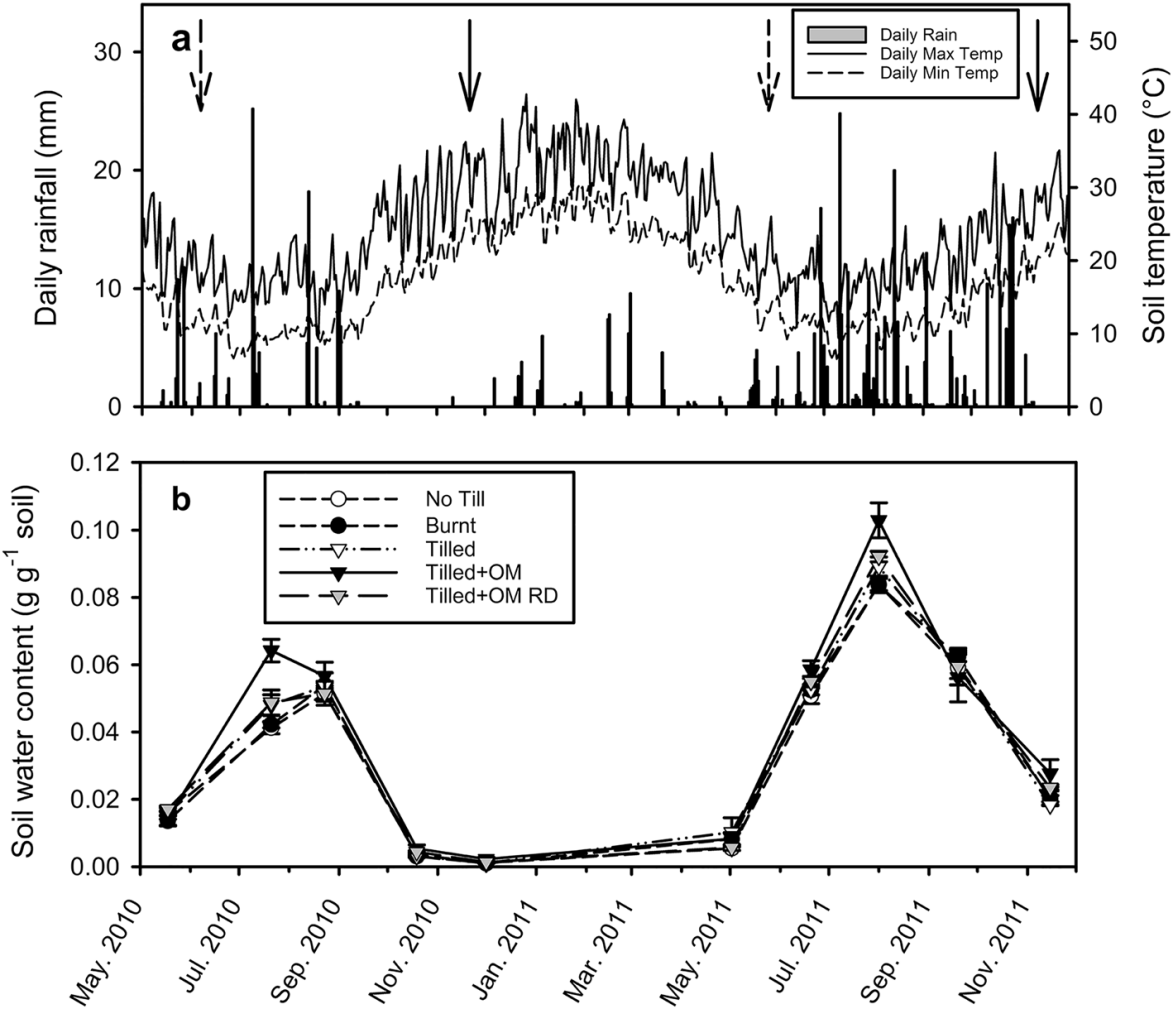
(**a**) Daily rainfall (bar graph, left y-axis) and daily soil minimum and maximum temperature at 5 cm depth (line graph, right y-axis) measured at the study site. (**b**) Soil water content (0–10 cm) at time of soil sample collection. Soil water content values are a mean of three field replicates, error bars are ± standard error of the mean. Dashed arrows indicate date of seeding (wheat, *Triticum aestivum*) and solid arrows indicate date of harvest. Nitrogen fertilizer was applied at seeding (28th May 2010 and 1st June 2011) and at crop emergence (12th July 2010 and 26th July 2011). *OM* organic matter, *RD* Run-Down.

Drylands, including semi-arid and arid regions, are important for agriculture, comprising one third of the world’s agriculturally productive lands and supporting nearly half of the global population^32,33^. Semi-arid agricultural soils in the grain-growing region of south-western Australia are generally acidic, sandy, low in soil OM content, and are annually exposed to soil temperatures greater than 40 °C during summer^34–36^. Rain in this region mainly falls during winter (the growing season), while agricultural soil is often left fallow during the dry summer (Fig. 1a). Soil NH_4_^+^ concentrations are usually less than 5 µg N g^−1^ dry soil except for short periods when NH_4_^+^-based fertilizers are applied to agricultural soil during the winter growing season^35,36^. Archaeal ammonia oxidizers would be expected to have greater abundance than AOB in these soils, due to their better resistance to elevated temperatures, low pH and low NH_4_^+^ availability^18,26,37^. Studies of surface soils in this region however have shown that AOB are similar to or more abundant than AOA^14,17,38,39^. As these studies sampled soil at one point in time, it is not yet clear whether this dominance of AOB occurs across all seasons, and how AOB and AOA populations vary dynamically with time in response to soil and environmental conditions.

Here we aimed to understand how soil OM that had been built by agricultural management practices in one semi-arid soil affected *amoA* gene abundance, and whether these interacted with season to modify population sizes of ammonia oxidizers. The overall objective of this two-year field-based study was to assess the temporal population dynamics of ammonia oxidizers so as to better understand potential N loss mechanisms in semi-arid environments dominated by winter rainfall. Specifically, this study investigated: (i) seasonal variation in ammonia oxidizer abundance; (ii) which soil and environmental factors regulated the relative abundances of AOB and AOA; and (iii) whether increased soil OM modified seasonal variation in ammonia oxidizer abundance. Five field-based OM treatments were chosen that were designed to alter soil C and N availability: no tillage (“No Till”), no till with burnt stubble (“Burnt”), tillage (“Tilled”), tillage with OM additions (“Tilled + OM”), and tillage with OM additions that ceased four years before this study commenced (“Tilled + OM Run-Down”). We hypothesized that there would be a greater abundance of AOB than AOA, AOB abundance would vary temporally in response to dissolved OM, and increasing soil OM would increase AOA relative to AOB throughout the year.

## Results

### Environmental conditions

Daily rainfall over the study period ranged from 0 to 25.2 mm (Fig. 1a). Growing season rainfall (April–October) was greater in 2011 (283 mm) than in 2010 (144 mm). Daily minimum soil temperature at 5 cm depth ranged from 6.6 °C in July 2011 to 30.4 °C in January 2011 (Fig. 1a). Daily maximum soil temperature at 5 cm depth ranged from 9.2 °C in August 2010 to 41.7 °C in January 2011 (Fig. 1a).

Soil water content at the time of soil collection followed a similar pattern to rainfall, increasing during the winter growing season (May–November) and at a minimum during summer (mean across OM treatments < 0.01 g g^−1^ dry soil; October 2010–May 2011; Fig. 1b). Mean maximum soil water content across OM treatments at sampling was greater in 2011 (0.09 g g^−1^ dry soil in August) than in 2010 (0.05 g g^−1^ dry soil in July–August; *p* < 0.0001). Tilled + OM soil had greater soil water content than the other OM treatments on two occasions in winter (July 2010, August 2011; *p* < 0.01), and greater soil water content than the No Till, Burnt and Tilled soils on one occasion in late spring (November 2011; *p* < 0.05; Fig. 1b).

### Dissolved organic C and microbial biomass C

**Figure 2:**
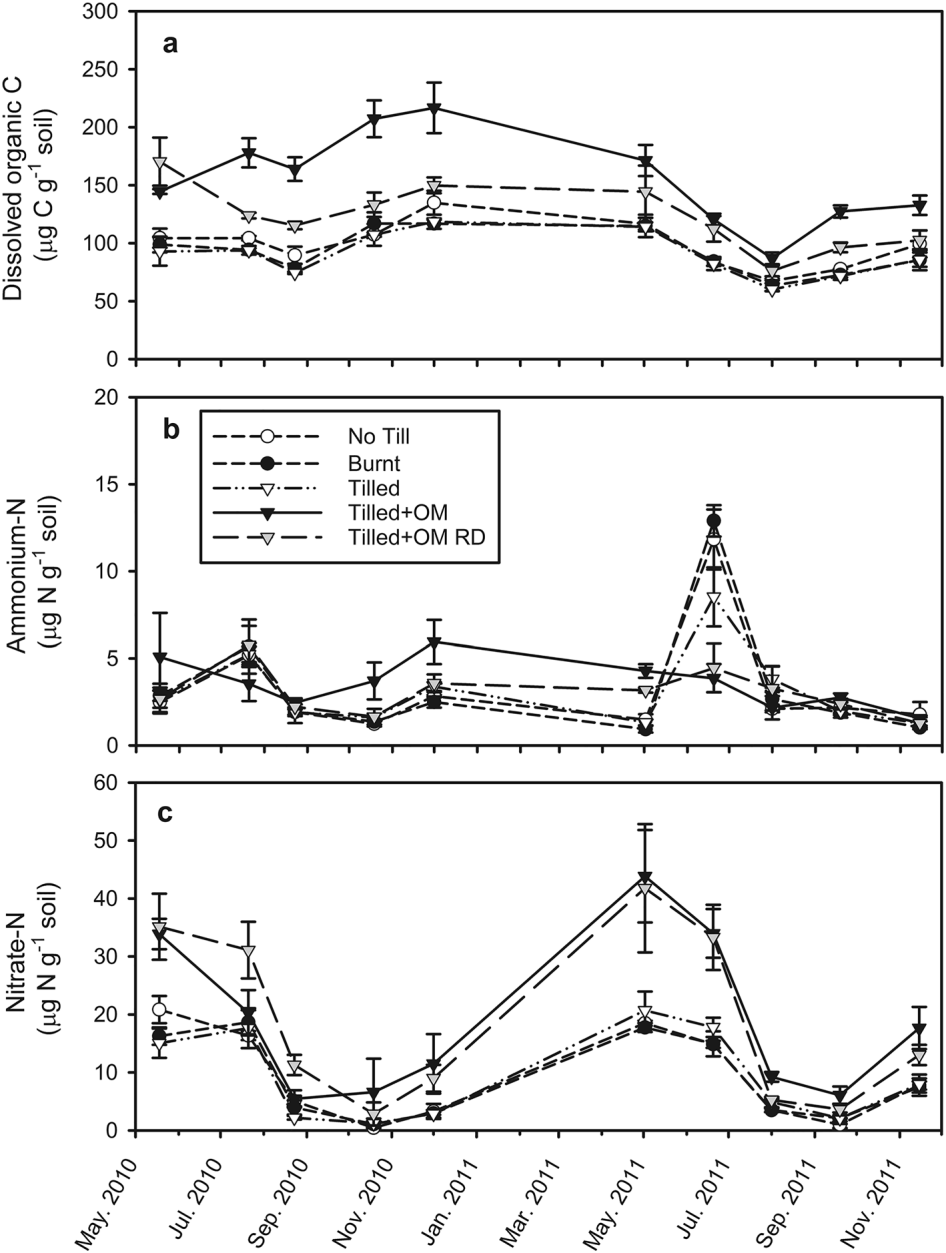
Change in (**a**) dissolved organic carbon; (**b**) ammonium; and (**c**) nitrate in soil (0–10 cm) through time, expressed per gram of dry soil. Values are a mean of three field replicates, error bars are ± standard error of the mean. Nitrogen fertilizer was applied at seeding (28th May 2010 and 1st June 2011) and at crop emergence (12th July 2010 and 26th July 2011). Legend is the same for all panels. *OM* organic matter, *RD* Run-Down.

**Figure 3:**
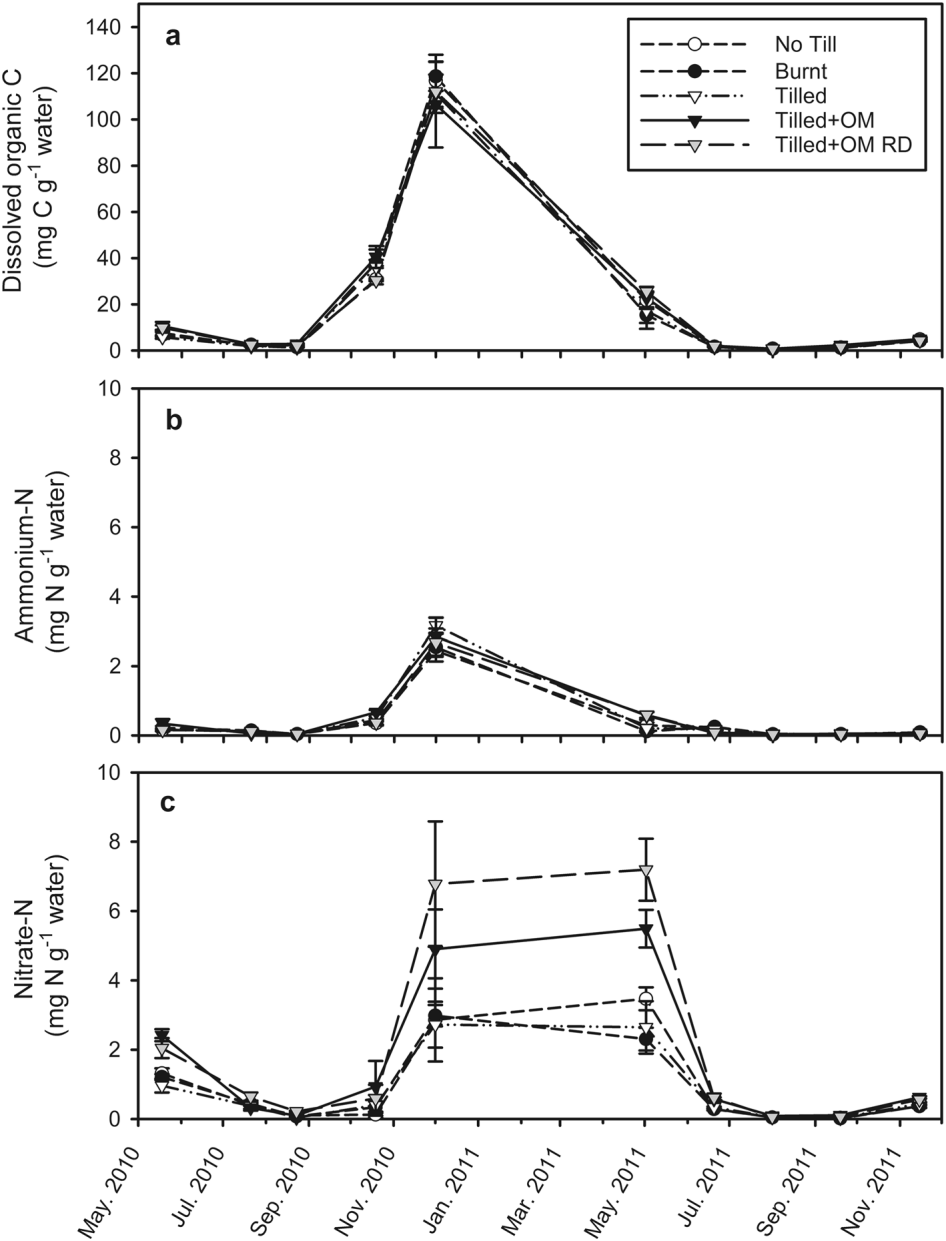
Change in (**a**) dissolved organic carbon; (**b**) ammonium; and (**c**) nitrate in soil (0–10 cm) through time, expressed per gram of soil water. Values are a mean of three field replicates, error bars are ± standard error of the mean. Legend is the same for both panels. Nitrogen fertilizer was applied at seeding (28th May 2010 and 1st June 2011) and at crop emergence (12th July 2010 and 26th July 2011). *OM* organic matter, *RD* Run-Down.

Dissolved organic matter (DOM) was analyzed as dissolved organic C (DOC). Mean DOC ranged from 60 to 217 µg C g^−1^ dry soil (Fig. 2a) and < 1 to 119 mg C g^−1^ water (Fig. 3a). During the dry summer months mean DOC concentration in soil solution averaged 113 mg C g^−1^ water (December 2010) compared to during winter where it is diluted to below 26 mg C g^−1^ water (May–September; Fig. 3a). However, DOC per gram of dry soil was at a maximum in December 2010 (summer fallow) and at a minimum in August 2011 (winter, mid-growing season; Fig. 2a). Although DOC in soil solution was statistically different between some OM treatments on each sampling date, the range between these treatments was very small (maximum statistically significant difference was 1.1 mg C g^−1^ water between Tilled and Tilled + OM soils on 19th Sep 2011; *p* < 0.001) compared to the variation of DOC over time. Dissolved organic C in Tilled + OM soil was greater than No Till, Burnt and Tilled soils at all sampling times (*p* < 0.01), and was greater than Tilled + OM Run-Down from July–December in 2010 (mid-winter to mid-summer), and from September–December in 2011 (spring to mid-summer; *p* < 0.01; Fig. 2a). Dissolved organic C in No Till, Burnt and Tilled soils were not different from each other (*p* > 0.05).

Mean microbial biomass C (MBC) ranged from 46 to 489 µg C g^−1^ dry soil (Supp. Fig. 1a). Microbial biomass C followed similar patterns with time in all OM treatments, but the Tilled + OM soil had significantly greater MBC than the other OM treatments (*p* < 0.05), except in May 2010 (autumn) and June 2011 (winter).

### Inorganic N and potentially mineralizable N

Total inorganic N concentrations (NH_4_^+^  + NO_3_^−^) were dominated by NO_3_^−^ (mean of 69%). Mean NH_4_^+^ concentrations ranged from < 1 to 3 mg N g^−1^ water (Fig. 3b) and < 1 to 13 µg N g^−1^ dry soil (Fig. 2b) while mean NO_3_^−^ concentrations ranged from < 1 to 7 mg N g^−1^ water (Fig. 3c) and < 1–44 µg N g^−1^ dry soil (Fig. 2c).

Mean NH_4_^+^ in soil solution peaked in summer when soil water content was lowest (December 2010; *p* < 0.0001; Fig. 3b). However, mean NH_4_^+^ per gram of dry soil in No Till, Burnt and Tilled soils peaked in winter (July 2010 and 2011), coinciding with N fertilizer inputs (Fig. 2b). Similar to DOC, NH_4_^+^ in soil solution was statistically different between some OM treatments on each sampling date, but these differences were small compared to seasonal variation in soil solution NH_4_^+^ (maximum statistically significant difference was 0.7 mg N g^−1^ water between Burnt and Tilled + OM RD soils on 20 Jun 2011; *p* < 0.0001). Surprisingly, NH_4_^+^ concentrations in the Tilled + OM soil did not respond to N fertilizer inputs, however NH_4_^+^ concentrations in Tilled + OM soil were greater than the other OM treatments during summer (October 2010–May 2011; *p* < 0.05).

Nitrate in soil solution was on average 816% greater in December 2010 (summer) and May 2011 (autumn) than at other times of the year (*p* < 0.05; Fig. 3c). Nitrate concentration in soil solution decreased significantly at the beginning of autumn growing season rains (May–June 2011; *p* < 0001 for all OM treatments). Soil NO_3_^−^ concentrations in all OM treatments also increased from the time of harvest by up to 1069% (*p* < 0.0001), in response to a series of summer rainfall events (compare Figs. 1a, 3b). Soil NO_3_^−^ peaked at the start of each growing season, and then declined to a minimum by the end of winter (around September; *p* < 0.0001; Fig. 2c). Mean NO_3_^−^ concentrations in Tilled + OM soil and Tilled + OM Run Down were greater than the other OM treatments in soil (*p* < 0.001) and in soil water over summer/autumn (May and December; *p* < 0.05).

Mean potentially mineralizable N (PMN) ranged from 10 to 98 µg N g^−1^ dry soil (Supp. Fig. S1b). Potentially mineralizable N in Tilled + OM soil peaked in August 2010 (late winter), approximately three months after OM additions to this treatment, and remained greater than the other OM treatments from July 2010 (winter) until December 2010 (summer; *p* < 0.05). Mean PMN in No Till, Burnt, Tilled and Tilled + OM Run-Down soils did not vary with time or between OM treatments from October 2010–November 2011 (spring 2010–spring 2011; *p* > 0.05).

### Ammonia-oxidizer gene abundance

Archaeal *amoA* gene abundance was below detection limits for 96% of samples (144 out of 150 samples; data not shown). Of the six samples in which AOA were detected, five were from No Till soil at different time points across all seasons sampled, and one was from the Tilled + OM Run-Down soil in June 2011. Detected archaeal *amoA* gene abundances ranged from 1.16 × 10^5^ to 3.25 × 10^7^ gene copies g^−1^ dry soil.

**Figure 4:**
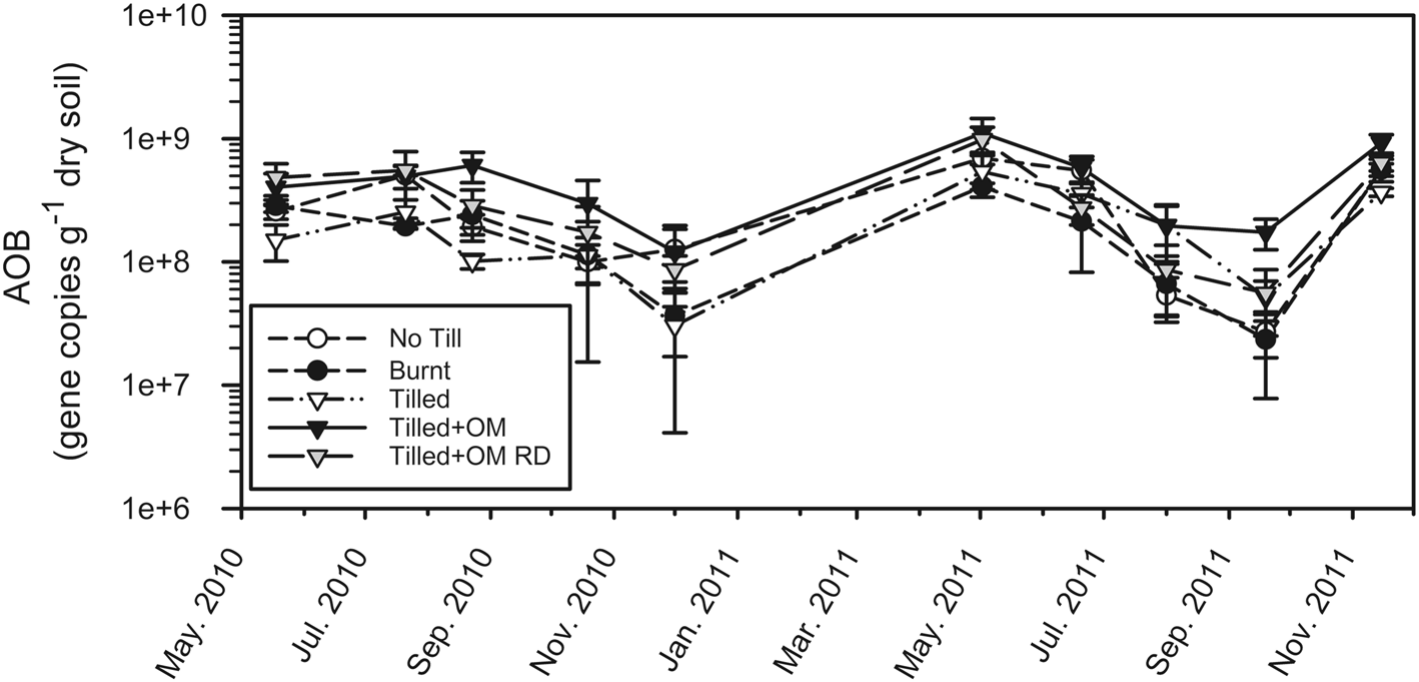
Change in soil (0–10 cm) bacterial *amoA* gene abundance (AOB) through time. Values are a mean of three field replicates, error bars are ± standard error of the mean. Note log scale of y-axis. *OM* organic matter, *RD* Run-Down.

Bacterial *amoA* gene abundance ranged from 2.35 × 10^7^ to 1.11 × 10^9^ gene copies g^−1^ dry soil (Fig. 4), and was detected in all samples. AOB varied seasonally in a similar way in each OM treatment. Bacterial *amoA* gene abundance generally decreased from the middle to the end of each growing season and was at a minimum in spring to early summer. Bacterial *amoA* gene abundance increased on average by 839% over summer to a maximum at the start of the growing season (from Dec 2010 to May 2011; *p* < 0.0001). When averaged across seasons, Tilled + OM soil had greater bacterial *amoA* gene abundance (mean of 4.91 × 10^8^ gene copies g^−1^ dry soil) than the other OM treatments (*p* < 0.05), and Tilled + OM Run-Down had greater bacterial *amoA* gene abundance (mean of 3.63 × 10^8^ gene copies g^−1^ dry soil) than Burnt and Tilled soils (means of 2.15 × 10^8^ and 2.17 × 10^8^ gene copies g^−1^ dry soil respectively; *p* < 0.05), but was not different from No Till soil (mean of 3.07 × 10^8^ gene copies g^−1^ dry soil; *p* > 0.05).

### Relationships between bacterial amoA gene abundance and other variables

Separate linear regressions of each variable were better related to bacterial *amoA* gene abundance (had the lowest Akaike Information Criteria) than multiple linear regressions using more than one variable. Bacterial *amoA* gene abundance had significant positive relationships with DOC per gram of soil (*p* < 0.01), MBC (*p* < 0.01) and NO_3_^−^ (*p* < 0.0001; Table 1; Supp. Fig. 2), but no relationship with cumulative 30-day rainfall, mean daily maximum or minimum soil temperature in the 30 days prior to sampling, soil water content at collection, NH_4_^+^ (in soil or in soil water), NO_3_^−^ in soil water, DOC in soil water or PMN (*p* > 0.05). The potential relationship between AOB and each significantly correlated variable was calculated by multiplying the coefficient by the maximum value measured for that variable. Variables with linear regression coefficients in order of greatest to least correlation to AOB were DOC, MBC, total inorganic N and NO_3_^−^.

**Table 1:** Significant linear regression results for response of logged bacterial *amoA* gene abundance to each soil and environmental variable separately.

Predictor	Significance level	Coefficient	Standard error of coefficient	Intercept	Standard error of intercept
log DOC	p = 0.0029	1.169	0.386	5.853	0.784
log MBC	p = 0.0012	0.744	0.226	6.665	0.475
sqrt NO_3_^−^	p < 0.0001	0.218	0.027	7.528	0.096
Rainfall	p = 0.9690				
Max. Temp.	p = 0.5436				
Min. Temp.	p= 0.8774				
Soil water	p = 0.1417				
log DOC_WC	p = 0.3186				
log NH_4_^+^	p = 0.3633				
log NH_4_^+^_WC	p = 0.1867				
sqrt NO_3_^−^_WC	p = 0.0510				
log PMN	p = 0.7610				

Regression coefficients are only reported for significant relationships.

*sqrt* square root transformed, *log* log transformed, *Rainfall*, cumulative rainfall of 30 days prior to sampling, *Max. Temp.* mean daily maximum soil temperature at 5 cm depth during 30 days prior to sampling, *Min Temp* mean daily minimum soil temperature at 5 cm depth during 30 days prior to sampling, *Soil Water* soil water content at time of collection, *DOC* dissolved organic carbon per gram of soil, *DOC_WC* dissolved organic carbon per gram of soil water, *MBC* microbial biomass carbon per gram of soil, *NH*_*4*_^+^ ammonium per gram of soil, *NH*_*4*_^+^*_WC* ammonium per gram of soil water, *NO*_*3*_^*−*^ nitrate per gram of soil, *NO*_*3*_^*−*^*_WC* nitrate per gram of soil water, *PMN* potentially mineralizable nitrogen per gram of soil.

**Figure 5:**
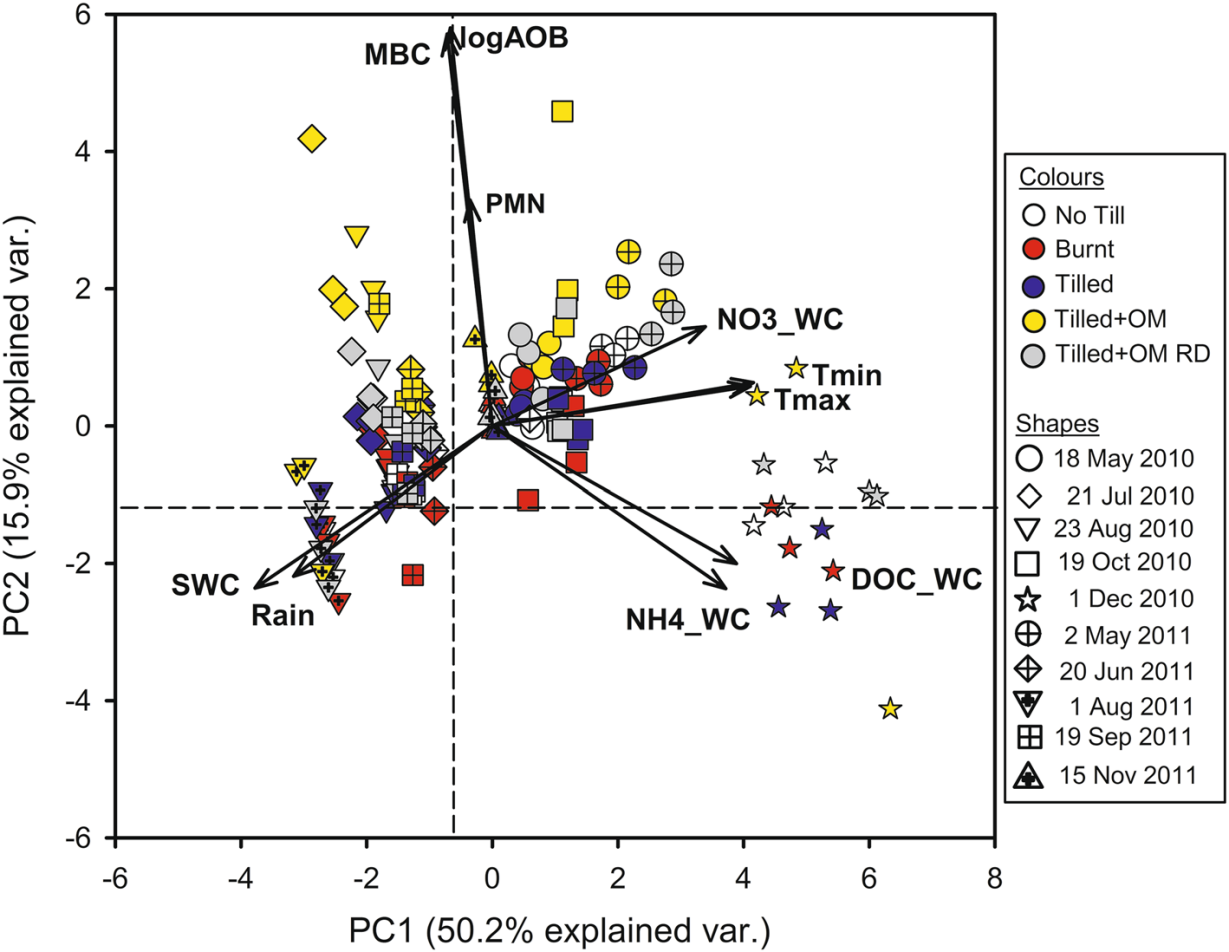
Principal component analysis biplot of principal components 1 (PC1) and 2 (PC2). *logAOB* logged bacteria *amoA* gene abundance, *Rain* cumulative rainfall over 30 days prior to sample collection, *SWC* soil water content at collection, *Tmax and Tmin* mean daily maximum and minimum soil temperature at 5 cm depth respectively over 30 days prior to sample collection, *MBC* microbial biomass carbon per gram of soil, *DOC_WC* dissolved organic carbon per gram of soil water, *NH4_WC* ammonium per gram of soil water, *NO3_WC* nitrate per gram of soil water, *PMN* potentially mineralizable nitrogen per gram of soil, *OM* organic matter, *RD* Run-Down. Symbol colours represent field organic matter treatments and symbol shapes represent sampling date.

Principal component analysis (PCA) showed that samples from the same collection date generally grouped across the biplot of principal components 1 (PC1) and 2 (PC2), roughly in line with PC1 as rainfall, soil water content and soil temperature varied (and consequently DOC, NH_4_^+^ and NO_3_^−^ in soil water varied; Fig. 5, collection dates shown by symbol shapes). Samples from the Tilled + OM soil, and Tilled + OM Run-Down to a lesser extent, had greater scatter across the biplot in line with PC2, compared to the No Till, Burnt and Tilled soils, which were grouped towards positive scores of PC2 (Fig. 5, OM treatments shown by symbol colours).

Five principal components were required to explain greater than 90% of the variance in soil biochemical and environmental properties (Supp. Table 1). PC1 explained 50.2% of total variance in the data (Supp. Table 1), and separated most strongly cumulative 30-day mean minimum and maximum soil temperatures (positive loadings) from cumulative 30-day rainfall and soil water content at collection (negative loadings; Fig. 5; Supp. Table 2). Otherwise, PC1 separated DOC in soil water, NH_4_^+^ in soil water and NO_3_^−^ in soil water (positive loadings near minimum and maximum temperature) from MBC, PMN and logged AOB gene abundance (with slightly negative loadings). PC2 explained 15.9% of total variance in the data (Supp. Table 1), and separated cumulative 30-day rainfall, soil water content at collection, DOC in soil water and NH_4_^+^ in soil water (negative loadings) from MBC, logged AOB gene abundance, and PMN (most strongly negative loadings; Fig. 5; Supp. Table 2). Otherwise, cumulative 30-day mean minimum and maximum temperatures and NO_3_^−^ in soil water were grouped together on PC2 (slightly positive loadings).

## Discussion

Ammonia-oxidizing bacteria rather than AOA appear to regulate nitrification processes in the surface layer of this semi-arid agricultural soil across all seasons. This agrees with previous studies from this region^14,38,39^, but is in contrast to other semi-arid environments and agricultural soils, which have found that AOA are often dominant to AOB, particularly in soils with low pH^23,39–41^. Here, we hypothesize that AOB were present but AOA were not detectable in the surface soil (96% of samples were undetected) as a consequence of the differing ecophysiologies and trace mineral requirements of each microorganism. Electron transfer enzymes in AOA are based on copper, in contrast to the iron-dependent ammonia oxidizing systems of AOB^13^. Soils in this Western Australian semi-arid region are ancient and highly weathered, so are rich in iron but deficient in copper^42,43^. Jenkins et al.^14^ observed a significant relationship between AOA and copper content in agricultural soils across Australia, suggesting that copper limitation of AOA is an important factor regulating niche differentiation between ammonia oxidizing organisms in soils in this region. Consequently, AOB is a target group for decreasing N losses, via leaching and denitrification, and improving N use efficiency in semi-arid regions containing highly weathered soils.

Seasonal variation in AOB abundance was primarily related to substrate concentration in pore water. Dissolved organic matter (here measured as DOC) and NH_4_^+^ in soil solution showed clear peaks in late winter at the end of the crop growing season (Fig. 3a,b), before an increase in AOB abundance during summer and autumn fallow and subsequent production of NO_3_^−^ (Figs. 3c, 4). Mineralization is inferred to be stimulated by crop plants (the source of DOM), particularly at the end of the growing season. Active nitrification and rapid depletion of NH_4_^+^ pools follows, as indicated by NO_3_^−^ dominating inorganic N pools in this soil (Figs. 2, 3)^35^. However as hypothesized, NH_4_^+^ concentrations in soil were not statistically related to abundance of AOB, although DOC, MBC and NO_3_^−^ concentrations in soil were. This was expected because soil NH_4_^+^ concentration (NH_4_^+^ pool size) is generally not a good indicator of NH_4_^+^ substrate availability or the activity of ammonia oxidizers^12^. Changes in soil solution volume in this semi-arid soil appear to have as much of an effect on substrate availability to microorganisms as the production of those substrates, through the concentrating effect of soil drying (i.e. physically locating the substrate into pores and water films where microorganisms are located).

The poor relationship between bacterial *amoA* gene abundance and rainfall, soil temperature, and soil water content was unexpected. Other authors have observed that climate variables can be as important, if not more, than agricultural management effects on soil and microbial processes in semi-arid soils^44,45^. For example, Wang et al.^41^ showed that AOB abundance was more sensitive to short-term N inputs and warming than AOA in a semi-arid grassland^26^. The lack of a relationship between AOB abundance and environmental conditions in the present study might be explained by a disconnect between longer-term population dynamics and shorter-term environmental events. Although directly influenced by environmental conditions such as soil water and temperature, abundances of functional genes are less dynamic than many environmental and edaphic properties, so functional gene abundances are likely to reflect the longer-term variation of those properties^46^. There was no linear correlation between AOB and climate characteristics, however the PCA showed that the variation in the AOB abundance, soil properties and climate data was partly explained by rainfall, soil water content and temperature, and clearly grouped by sampling date (Fig. 5, PC1 in Supp. Table 2). These findings suggest that seasonal dynamics of AOB populations are not as yet well understood, but are due to many interacting factors that will consequently influence risk of N loss through the production of NO_3_^−^.

Organic matter inputs to soil have positively but indirectly influenced bacterial *amoA* gene abundance (irrespective of the time of year) due to the enhanced supply of either C or N substrates. The effect of soil OM content on AOB in the present study did not support our hypothesis and varied from observations in other semi-arid soils. For example, whilst bacterial *amoA* gene abundance was positively correlated with soil OM in our study, it was negatively correlated with soil OM in summer and winter over six years in a semi-arid shrubland soil^26^. This negative correlation would be expected to occur if soil OM stimulates heterotrophic microorganisms to compete more successfully for NH_4_^+^, decreasing N substrate availability and thus growth of autotrophic ammonia oxidizers. The positive effect of increased soil OM pools on AOB abundance in the present study could be attributed to the associated increases in N supply through DOM to autotrophic ammonia oxidizers. Previous work on the same soil showed that OM inputs increased gross N mineralization, immobilization as well as nitrification rates^47^, suggesting that increased soil OM stimulates both heterotrophic and autotrophic microbial populations and activity.

Bacterial ammonia oxidizer growth during summer fallow is likely an important factor determining increases in soil NO_3_^−^ pools and consequently risk of N loss in this semi-arid soil. Soil NO_3_^−^ pools are a balance of inputs and outputs. Nitrate inputs into the surface soil (0–10 cm) appear to continue as summer progresses with maintenance of NO_3_^−^ concentrations in soil solution (Fig. 3c) and increased total soil NO_3_^−^ (Fig. 2c). Nitrate inputs over the dry season has also been observed in the same and similar semi-arid soils in response to summer rainfall events that stimulate N mineralization^35,36,48^. In contrast to inputs, outputs of NO_3_^−^ in this region are likely to be low during summer fallow, with plant uptake minimal or non-existent due to the absence of plants, and it is not until the start of the growing season rains that any significant deep drainage and NO_3_^−^ leaching usually occurs^49^. A significant decrease in concentration of NO_3_^−^ in soil solution was observed in the present study in early autumn (Fig. 3c), likely due to leaching and dilution by rainwater. Correlation of bacterial *amoA* gene abundance and NO_3_^−^ pool size in soil suggests that increases in NO_3_^−^ during summer are not due merely to N mineralization flushes and lack of inorganic N uptake, but also due to growth of the AOB population, strongly coupled to activity of nitrite-oxidizing bacteria. Bacterial *amoA* gene abundance was less in summer than in winter in another semi-arid soil from Israel, which was attributed to the sensitivity of AOB to high temperatures, at that site ranging from 23 to 35 °C in summer^26^. By contrast, bacterial *amoA* gene abundance in the present study continued to increase over summer, even though soil temperatures exceeded 40 °C (Fig. 1a). This may be because AOB were sampled more often, so finer scale population dynamics could be observed in this soil. An alternative explanation is that AOB in the present study region have adapted to periods of high temperature and low water availability, allowing them to survive then continue to grow when conditions become favourable, even if only briefly due to transient water pulses from summer rainfall events.

Predicted changes in climate for this region are likely to exacerbate the effects of AOB population dynamics and NO_3_^−^ production, particularly during summer fallow. Since the 1970s, mean annual rainfall in Southern Hemisphere semi-arid regions has been decreasing, especially during autumn and winter at the start of the growing season^50,51^. Summer rainfall events however are increasing^52^, which will likely enhance inorganic N supply through OM decomposition when there is no plant N uptake from fallow soil^48,53^. This inorganic N is then at risk of loss if it is in excess of microbial demand and is subjected to nitrification. Managing undesirable losses of N from soil in response to summer rainfall events change in this semi-arid region is likely to become increasingly challenging, unless the activity and growth of the AOB population can be controlled (or excess inorganic N utilized by plants or soil microorganisms before it is lost from the soil).

In conclusion, seasonal variation of bacterial *amoA* gene abundance in this soil is contributing to NO_3_^−^ production and therefore the risk of N loss, especially as summer fallow progresses. Archaea however are unlikely to be influential drivers of ammonia oxidation as archaeal *amoA* gene abundances were predominantly below detection limits in the surface layers, possibly due to copper limitation of archaeal ammonia oxidation enzyme systems. Increased soil OM levels by additional crop residue inputs positively influenced bacterial *amoA* gene abundances, but did not modify seasonal variation in ammonia oxidizer abundance. Increases in AOB abundance over summer fallow followed a pulse of DOM and NH_4_^+^ in soil solution at the end of the winter growing season. As expected, bacterial *amoA* gene abundance was not statistically related to NH_4_^+^ concentration in soil, but surprisingly was also not related to soil water content, 30-day rainfall or mean soil temperature, suggesting that ammonia oxidizer populations in these semi-arid soils are regulated by longer-term changes in climate and substrate supply.

## Methods

### Study site and soil

Temporal variation of ammonia-oxidizing populations was examined at a field research site with arable management treatments to alter soil OM without the confounding effects of climate and soil type. The site is in the northern grainbelt of Western Australia (30.00° S, 116.33° E), with a Mediterranean semi-arid climate. Mean annual rainfall is 291 mm and mean monthly temperatures range from 5.8 to 35.3 °C [1997–2016 data, from a weather station at Dalwallinu (30.28° S, 116.67° E); Commonwealth of Australia Bureau of Meteorology, http://www.bom.gov.au/climate/data]. Rainfall and soil temperature data was collected at the site (Fig. 1a) using a Tipping Bucket Raingauge Model TB4 (Hydrological Services, Liverpool, NSW, Australia) and CS Model 107 Temperature Probes (Campbell Scientific, Logan, Utah, USA). The soil is a sand (92% sand, 2% silt, 6% clay), classified as a Basic Regolithic Yellow-Orthic Tenosol^54^ or a Haplic Arenosol^55^.

### Experimental design and soil collection

The site had a three-year rotation of lupin (*Lupinus angustifolius*)—wheat (*Triticum aestivum*)—wheat since 2003, with one rainfed crop each winter. The site had three randomized blocks that each included five OM treatment plots (80 m by 10 m). The five OM treatments were: (i) no tillage with full stubble retention, seeded with knife point tines to 15 cm depth (No Till); (ii) no tillage with burnt stubble (Burnt); (iii) soil tilled with offset disks to 10 cm depth prior to seeding (Tilled); (iv) tilled soil loaded with additional OM (Tilled + OM); and (v) Tilled + OM Run-Down, where additional OM was applied between 2003 and 2006 and then ceased. OM was applied at 20 t ha^−1^ to both Tilled + OM and Tilled + OM Run-Down plots in 2003 (barley straw) and 2006 (canola chaff), and to Tilled + OM plots in 2010 (oat chaff). OM was applied after each lupin crop, and before seeding of wheat. Seven years after the study commencement, soil organic carbon (SOC) contents ranged from 0.91 to 1.38%, and total N contents ranged from 0.09 to 0.13% (Table 2).

**Table 2:** Properties of field organic matter treatments (0–10 cm depth) at start of present study, 7 years after treatments were imposed.

	No Till	No till burnt stubble	Tilled	Tilled + OM	Tilled + OM Run-Down
Bulk density (g cm^−3^)^#^	1.58 ± 0.04^ab^	1.60 ± 0.01^ab^	1.63 ± 0.03^b^	1.42 ± 0.05^a^	1.40 ± 0.08^a^
pH (CaCl_2_)^§^	6.1 ± 0.1^a^	6.2 ± 0.1^a^	6.2 ± 0.2^a^	6.2 ± 0.2^a^	6.3 ± 0.0^a^
EC (dS m^−1^)^‡^	0.09 ± 0.008^a^	0.10 ± 0.004^a^	0.08 ± 0.004^a^	0.17 ± 0.018^b^	0.17 ± 0.013^b^
Total carbon (%)^†^	0.94 ± 0.02^a^	1.05 ± 0.03^ab^	0.91 ± 0.02^a^	1.22 ± 0.15^ab^	1.38 ± 0.07^b^
Total nitrogen (%)^†^	0.09 ± 0.001^a^	0.10 ± 0.003^ac^	0.09 ± 0.002^a^	0.12 ± 0.010^bc^	0.13 ± 0.004^b^
C:N ratio	11.0 ± 0.30^a^	11.0 ± 0.06^a^	10.5 ± 0.05^a^	10.1 ± 0.39^a^	10.9 ± 0.26^a^
Copper (µg g^−1^)^∞^	0.54 ± 0.06^a^	0.51 ± 0.02^a^	0.51 ± 0.01^a^	0.54 ± 0.04^a^	0.54 ± 0.06^a^
Iron (µg g^−1^)^∞^	13.59 ± 1.41^a^	13.41 ± 2.11^a^	10.95 ± 1.78^a^	15.99 ± 2.41^a^	14.70 ± 0.23^a^

Values are a mean of three field replicates ± standard error of the mean. Organic matter treatments with the same letter are not significantly different (*p* > 0.05).

*OM* organic matter.

^#^Bulk density determined using the intact core method with 3 cores of 7.35 cm diameter by 10 cm depth^66^.

^§^pH was determined on air-dry soil in 0.01 M CaCl_2_ with a 1:5 soil:extract ratio, after shaking for 1 h, and while stirring the soil suspension^67^.

^‡^Electrical conductivity was determined on air-dry soil in water with a 1:5 soil:water ratio^67^.

^†^Total carbon and nitrogen were determined by high-temperature combustion of finely ground air-dry soil using an Elementar Vario MACRO CNS elemental analyzer (Hanau, Germany)^67^.

^∞^Trace elements copper and iron were determined by atomic absorption spectroscopy after extraction with diethylene-triamine-penta-acetic acid (DTPA) solution (ratio of 1:2) for 2 h^67^.

Two crops of winter wheat were grown during the present study, and fertilized at rates depending on expected growing season rainfall and projected potential yields (1.5–2.5 t ha^−1^ in 2010, 3–4 t ha^−1^ in 2011), as is standard practice in this region. In 2010, 60 kg ha^−1^ granular fertilizer (NPKS—10.2:12.0:11.2:6.0; N as (NH_4_)_2_SO_4_ and monoammonium phosphate) was applied at seeding (28th May), and 40 L ha^−1^ of liquid fertilizer (NPKS—32.0:0:0:0; N as urea and NH_4_NO_3_) was applied at crop emergence (12th July), for a total of 23.0 kg N ha^−1^ y^−1^. In 2011, 60 kg ha^−1^ solid and 20 L ha^−1^ liquid fertilizer were applied at seeding (1st June; NPKS and N form as above), and 50 L ha^−1^ liquid fertilizer was applied at emergence (26th July) for a total of 35.7 kg N ha^−1^ year^−1^. Harvest in 2010 was on 16th November, and in 2011 was on 14th November.

Soil was collected (0–10 cm depth) on ten occasions from each plot between May 2010 and November 2011. Within each plot, 25 cores (5.3 cm diameter × 10 cm depth) were sampled using a zigzag pattern. Subsamples of soil from each plot were frozen for DNA analysis of AOA and AOB on return from the field. Remaining soil was sieved to < 2 mm and stored field-moist at 4 °C. Soils were analysed for gravimetric soil water content and inorganic N at field water content. All soils were adjusted to 45 to 55% water holding capacity^56^, and then pre-incubated for 7 days at 25 °C before analysis for MBC, DOC and PMN. On the other sampling dates there was no need to first wet-up the soil.

### Microbial biomass C and dissolved organic matter

Microbial biomass C was measured using the chloroform fumigation–extraction method^56^. Fresh soil (10 g) was fumigated with chloroform (stabilized by amylene) under vacuum in the dark for 24 h at 25 °C. Fumigated samples, with replicate non-fumigated samples, were extracted by shaking for 1 h with 40 mL of 0.5 M K_2_SO_4_. Filtered extracts (Whatman No. 42) were frozen until further analysis. Fumigated and non-fumigated extracts were analysed using an OI Analytical Aurora 1030 Wet Oxidation TOC Analyser (College Station, TX, USA) for non-purgeable organic C. DOC values were determined using the results from non-fumigated extracts, as an indicator of DOM. DOC was expressed either as per gram of oven-dry soil, or as per gram of soil water, at the time of soil sampling. The amount of soil water was determined by measuring the gravimetric water content. MBC was calculated from the difference between fumigated and non-fumigated organic C, divided by a k_EC_ factor of 0.45^57^.

### Inorganic N analysis and potentially mineralizable N

Inorganic N was extracted from 20 g soil by shaking for 1 h with 80 mL of 0.5 M K_2_SO_4_. Filtered extract solutions (Whatman No. 42) were kept frozen until colorimetric analysis on a Skalar San Plus auto-analyzer (Breda, The Netherlands), using the modified Berthelot reaction for NH_4_^+^^58^ and the hydrazinium reduction method for NO_3_^−^^59^. Both NH_4_^+^ and NO_3_^−^ were expressed per gram of oven-dry soil and per gram of soil water as described above.

Potentially mineralizable N was determined by anaerobic incubation^60,61^. Fresh soil (20 g) was incubated in 80 mL of deionized water for 7 days at 40 °C. K_2_SO_4_ was added (6.97 g) to adjust the soil solution to 0.5 M K_2_SO_4_, then samples were shaken for 1 h. Replicate 20 g samples of non-incubated soil in 80 mL of 0.5 M K_2_SO_4_ were also shaken. Incubated and non-incubated samples were filtered (Whatman No. 42) then frozen until further analysis. Samples were analysed for NH_4_^+^ on an autoanalyser, as described above.

### Nucleic acid extraction and qPCR

In March 2014, DNA was extracted from 700 mg subsamples of field-moist soil^62^. The functional genes encoding bacterial and archaeal ammonia monooxygenase (*amoA*) were determined by qPCR. Bacterial *amo*A primers were amoA-1F (5′-GGGGTTTCTACTGGTGGT-3′), and amoA-2R (5′-CCCCTCKGSAAAGCCTTCTTC-3′), amplifying a fragment length of 491 bp^9^. Bacterial *amoA* gene abundance was quantified using an ABI 7500 Fast qPCR machine (Applied Biosystems, Carlsbad, CA, USA). Each 20 µL qPCR reaction contained 10 µL of Power SYBR Green PCR Master Mix (Applied Biosystems, Warrington, UK), 0.2 µL each of the specific forward and reverse primers at 10 µM, 2 µL of bovine serum albumin at 5 mg mL^−1^ (Ambion UltraPure BSA, Carlsbad, CA, USA), 2 µL of template DNA and 5.6 µL of water. Thermocycling conditions were: 95 °C for 10 min; then 40 cycles of 94 °C for 60 s, 56 °C for 60 s, 72 °C for 60 s and 78 °C for 60 s; followed by a melt curve to establish a single product amplification. Fluorescence data was collected at the 78 °C stage.

Archaeal *amoA* primers were Arch-amoAF (5′-STAATGGTCTGGCTTAGACG-3′) and Arch-amoAR (5′-GCGGCCATCCATCTGTATGT-3′) amplifying a fragment length of 635 bp^63^. Archaeal *amoA* gene abundance was quantified using an Applied Biosystems ViiA™ 7 (Carlsbad, CA, USA). Each 10 µL qPCR reaction contained 5 µL of Power SYBR Green PCR Master Mix (Applied Biosystems, Warrington, UK), 0.1 µL each of the specific forward and reverse primers at 10 µM, 1 µL of BSA, 1 µL of template DNA and 2.8 µL of water. Thermocycling conditions were: 94 °C for 10 min; then 40 cycles of 94 °C for 1 min, 52 °C for 1 min, 72 °C for 1 min and 78 °C for 1 min; followed by a melt curve to establish a single product amplification. Fluorescence data was collected at the 78 °C stage.

Standard templates used to determine gene copy numbers in the qPCR reactions were cloned plasmids, as described in Barton et al.^64^. Samples were tested over a series of dilutions to determine if there was inhibition, and further analysis was completed using the dilution that produced the highest copy number. Standard curves generated in each reaction were linear over four orders of magnitude for AOB (10^3^–10^6^ gene copies) and six orders of magnitude for AOA (10^3^–10^8^) with r^2^ values greater than 0.98. Amplification efficiencies ranged from 79 to 8% for AOA amoA quantification and 94–111% for quantification of AOB amoA.

### Statistical analysis

Statistical differences between OM treatments for the basic soil properties were determined using ANOVA with associated TukeyHSD post hoc tests in R version 3.1.0 (R Foundation for Statistical Computing, Vienna, Austria). Bacterial *amoA* gene abundance was initially log transformed before all statistical analyses. Statistical significances of OM treatment with time for each soil property were evaluated by mixed linear models estimated by restricted maximum likelihood (REML), using PROC MIXED in SAS version 9.3 (SAS Institute Inc., Cary, NC, USA), because replication was at the highest level (i.e. replicates were field plots). Random effects of field block and field block interacting with OM treatment were included in the model. For this analysis, NH_4_^+^ in soil and in soil water, PMN, MBC in soil and soil water and DOC in soil and soil water were also log transformed, while total inorganic N, NO_3_^−^ in soil and soil water and AOB abundance were square root transformed to ensure a normal distribution. When there was a significant interaction between OM treatment and date, and multiple pairwise comparisons were needed to support results statements, the reported P value is the maximum P value of the relevant pairwise comparisons.

Backwards multiple linear regression was also conducted using PROC MIXED. Random effects of field block and field block interacting with OM treatment were included in the model. The best regression model was evaluated by minimizing the Akaike Information Criteria^65^. PCA was carried out using prcomp in R version 3.1.0 with scaled and centred data.

### Experimental protocols

All plant experiments were in compliance with relevant institutional, national, and international guidelines and legislation.

## Supplementary Information

Supplementary Information.

## Data Availability

The datasets generated during and/or analysed during the current study are available from the corresponding author on reasonable request.
