# Supplementary material for: Seasonal dynamics of ammonia-oxidizing bacteria but not archaea influence soil nitrogen cycling in a semi-arid agricultural soil
Source: Sci Rep. 2022 May 4;12:7299. doi: 10.1038/s41598-022-10711-0 (PMC9068766; doi:10.1038/s41598-022-10711-0)
Supplement: Supplementary file 1 — Supplementary Information. [file 41598_2022_10711_MOESM1_ESM.pdf]

Supplementary Information:

Seasonal dynamics of ammonia-oxidizing bacteria but not archaea influence soil nitrogen cycling in a semi-arid agricultural soil

L. M. Fisk, L. Barton*, L. D. Maccarone, S.N. Jenkins, and D. V. Murphy

SoilsWest, UWA School of Agriculture and Environment, The University of Western Australia, 35 Stirling Highway, Crawley, WA 6009, Australia.

*Corresponding author. Tel.: +61 8 6488 2543

Email address: louise.barton@uwa.edu.au

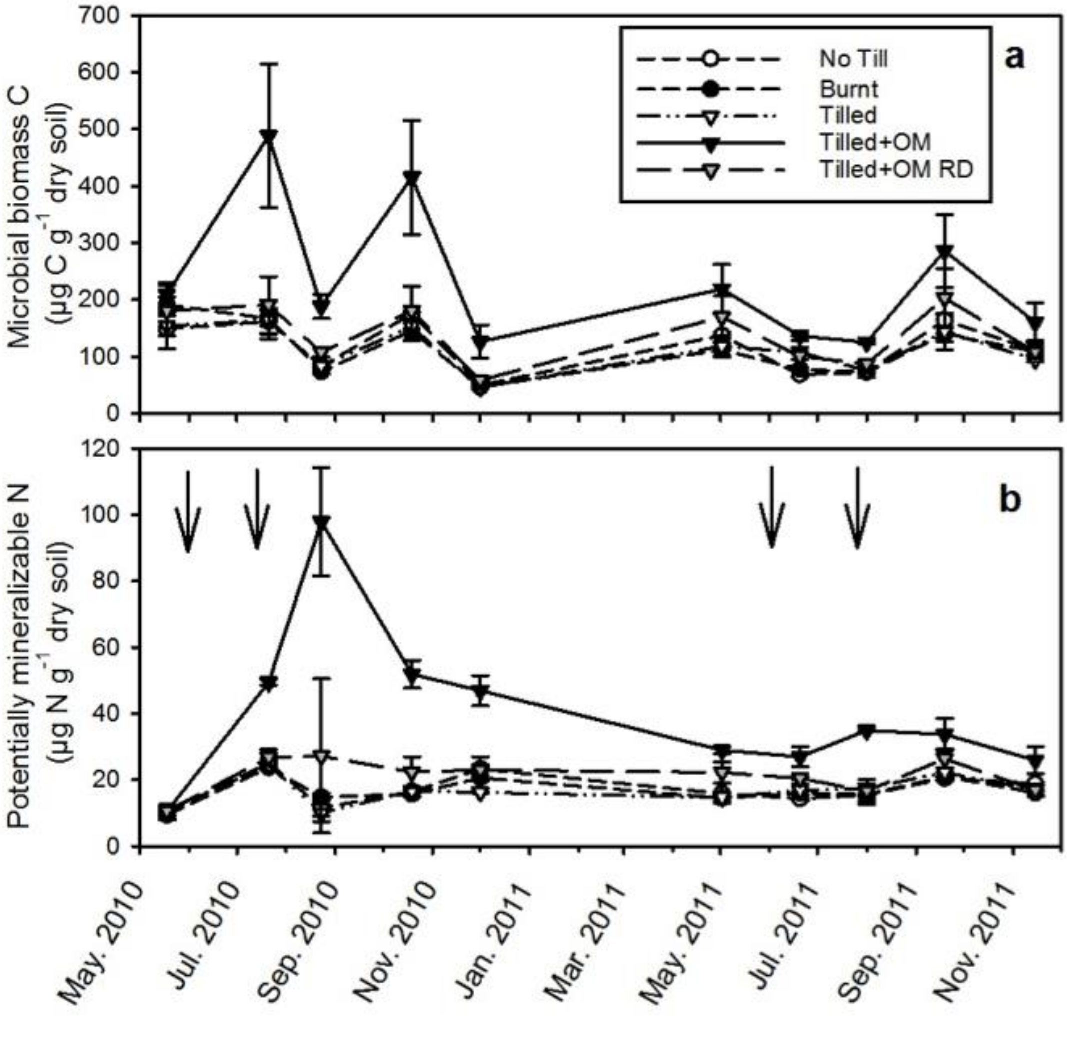

Supplementary Figure 1: Change in (a) microbial biomass carbon; and (b) potentially mineralizable nitrogen in soil (0–10 cm) through time. Values are a mean of three field replicates, error bars are ± standard error of the mean. Arrows indicate dates of N fertilization. Legend is the same for all panels. Legend abbreviations: OM, organic matter; RD, Run-Down.

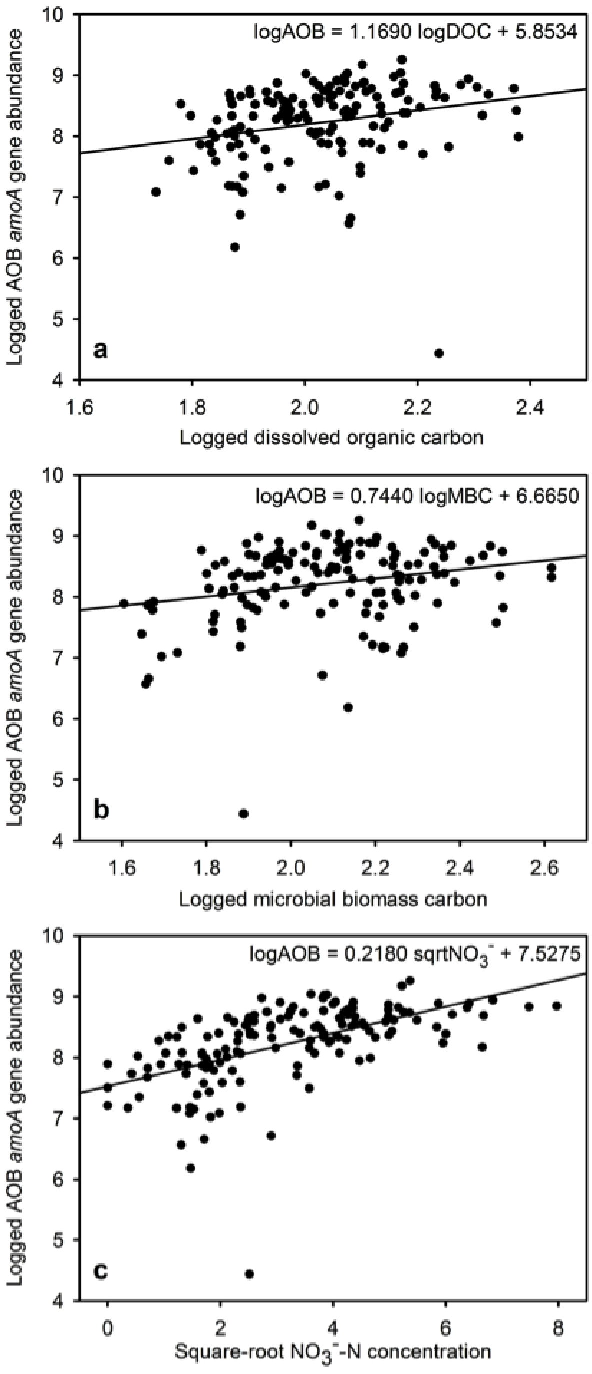

Supplementary Figure 2. Significant linear regression relationships between logged bacterial *amoA* gene abundance per gram of dry soil (logAOB) and (a) logged dissolved organic carbon per gram of soil (logDOC); (b) logged microbial biomass carbon per gram of soil (logMBC); and (c) square root transformed nitrate per gram of soil (sqrtNO_3^-).

Supplementary Table 1. Eigenvector loadings of principle components 1–5.

	**PC1**	**PC2**	**PC3**	**PC4**	**PC5**
Standard deviation	2.240	1.262	1.150	0.844	0.692
Variance (eigenvalue)	5.019	1.593	1.323	0.712	0.478
Proportion of variance	0.502	0.159	0.132	0.071	0.048
Cumulative proportion of variance	0.502	0.661	0.794	0.865	0.913

Supplementary Table 2. Loadings matrix (eigenvectors) for principle components 1–5. Abbreviations: Rain, cumulative rainfall of 30 days prior to soil collection; SWC, soil water content at collection; Min. Temp. and Max. Temp., mean daily minimum and maximum soil temperature at 5 cm depth of 30 days prior to soil collection; MBC, microbial biomass carbon per gram of soil; DOC_WC, dissolved organic carbon per gram of soil water; NH4_WC, ammonium per gram of soil water; NO3_WC, nitrate per gram of soil water; PMN, potentially mineralizable nitrogen per gram of soil; log AOB, logged bacterial *amoA* gene abundance per gram of soil.

	**PC1**	**PC2**	**PC3**	**PC4**	**PC5**
Rain	-0.317	-0.219	-0.083	-0.502	-0.442
SWC	-0.378	-0.236	-0.207	-0.191	0.189
Min. Temp.	0.416	0.063	0.145	-0.090	-0.363
Max. Temp.	0.411	0.058	0.112	-0.060	-0.457
MBC	-0.072	0.571	-0.355	0.458	-0.085
DOC_WC	0.389	-0.200	-0.278	-0.059	0.019
NH4_WC	0.372	-0.237	-0.311	-0.122	0.134
NO3_WC	0.339	0.145	-0.034	-0.333	0.608
PMN	-0.036	0.330	-0.685	-0.331	-0.164
log AOB	-0.067	0.580	0.382	-0.501	0.081
